# Surveying the Synthesis, Optical Properties and Photocatalytic Activity of Cu_3_N Nanomaterials

**DOI:** 10.3390/nano12132218

**Published:** 2022-06-28

**Authors:** Patricio Paredes, Erwan Rauwel, Protima Rauwel

**Affiliations:** Institute of Forestry and Engineering Sciences, Estonian University of Life Sciences, Kreutzwaldi 56/1, 51014 Tartu, Estonia; patricio.paredes@emu.ee (P.P.); erwan.rauwel@emu.ee (E.R.)

**Keywords:** Cu_3_N, nanostructures, synthesis, optical properties, photocatalysis

## Abstract

This review addresses the most recent advances in the synthesis approaches, fundamental properties and photocatalytic activity of Cu_3_N nanostructures. Herein, the effect of synthesis conditions, such as solvent, temperature, time and precursor on the precipitation of Cu_3_N and the formation of secondary phases of Cu and Cu_2_O are surveyed, with emphasis on shape and size control. Furthermore, Cu_3_N nanostructures possess excellent optical properties, including a narrow bandgap in the range of 0.2 eV–2 eV for visible light absorption. In that regard, understanding the effect of the electronic structure on the bandgap and on the optical properties of Cu_3_N is therefore of interest. In fact, the density of states in the d-band of Cu has an influence on the band gap of Cu_3_N. Moreover, the potential of Cu_3_N nanomaterials for photocatalytic dye-degradation originates from the presence of active sites, i.e., Cu and N vacancies on the surface of the nanoparticles. Plasmonic nanoparticles tend to enhance the efficiency of photocatalytic dye degradation of Cu_3_N. Nevertheless, combining them with other potent photocatalysts, such as TiO_2_ and MoS_2,_ augments the efficiency to 99%. Finally, the review concludes with perspectives and future research opportunities for Cu_3_N-based nanostructures.

## 1. Introduction

The production of nanoscale structures has attracted interest as they exhibit size and shape-dependent physical, electrical and chemical properties that are absent in their bulk counterparts [1,2,3]. Transition metal nitride nanostructures such as Co_2_N [4], TiN [5], OsN_2_ [6], Zn_3_N_2_ [7] and IrN_2_ [8] are rising as strong candidates for a variety of industrial applications, as they display excellent catalytic, electrochemical and optoelectronic properties. Transition metal nitrides simultaneously behave as ionic crystals, transition metals and covalent compounds [9]. They are therefore being investigated as potential contenders for electrochemical energy conversion, heterogeneous catalysis, fuel cells, batteries and supercapacitors [10,11,12,13,14]. Additionally, their optical absorption, electrical properties and tolerance to structural defects make them a new class of semiconductors of interest [15]. The most popular metal nitrides in the semiconductor family are GaN, AlGaN and InGaN, which produce blue light-emitting diodes (LEDs) and lasers [16,17,18]. Furthermore, semiconductors, such as Ta_3_N_5_ [19], InN [20,21] and InGaN [22,23], are also potential candidates for photocatalysis due to their simple chemical composition, tunable narrow band gap and high stability. However, the metals, i.e., In, Ta and Ga, are relatively rare in the Earth’s crust, and the price of indium has shot up by 900% in the last 10 years. Therefore, more sustainable metallic raw materials are required for industrial applications.

In that regard, Cu_3_N, composed of earth-abundant elements, is a potential candidate for industrial applications. Moreover, owing to its cost-effectiveness of production and environmental friendliness, it has gained importance. In addition, due to its physicochemical characteristics and tunable optical and electrical properties, Cu_3_N is the focus of several research investigations [24,25]. The shape and size-controlled synthesis of Cu_3_N nanoparticles is one of the main challenges today. The current synthesis methods usually involve the use of reactive nitrogen precursors, high pressure and temperature [26]. Synthesis techniques, such as thermal decomposition [27], electroplating [28], solvothermal [26], chemical vapor deposition (CVD) [29], radio-frequency (RF) and direct current (DC) magnetron reactive sputtering [30,31,32] have been employed for the synthesis of Cu_3_N thin films. For nanoparticle synthesis, several reports claim that solution-based synthesis approaches tend to offer a better way of controlling the morphology and properties of Cu_3_N nanoparticles [24,33] by carefully varying the synthesis conditions, i.e., time, temperature and precursors. Although Cu_3_N itself has been extensively studied during the last decade, there is still a significant disparity between the theoretical and experimental optical band gaps. Nevertheless, all these studies concede that Cu_3_N has a narrow bandgap with values ranging from 0.2 to 2.0 eV, exhibiting either metallic or semiconductor behavior [30,34,35]. In fact, the physical and chemical properties of the material not only depend on the synthesis conditions but also on the presence of dopants in the Cu_3_N lattice [36,37].

Cu_3_N has been introduced as a new class of materials for the next generation of photovoltaic and photocatalytic applications due to its high optical absorption, good electrical properties and p-type defect-tolerant semiconductor characteristics [38,39,40]. Photocatalysis is an advanced oxidation process in which illumination is used to separate excitons that are then transferred to the surface of the nanoparticles [41]. The oxidation–reduction reactions produce reactive oxygen species (ROS) that oxidize organic matter on the surface of the nanomaterial leading to its degradation. The mechanism is explained in detail in Section 4 of this review. According to Cheng et al., transition metal nitrides are capable of reducing the overpotential or activation energy for photocatalytic reactions by providing additional active sites that promote electron–hole separation [39]. Furthermore, improvement in the catalytic yield of Cu_3_N has been achieved by doping with transition metal ions, such as Au, Ni, Cr, Fe and Co, at the interstitial sites of the cubic structure. In addition, some metallic nanoparticles such as Ag, Au and Cu display localized surface plasmon resonance (LSPR) that enhances their optical and electrical properties. In general, metal–semiconductor junctions tend to enhance the physicochemical performances of the heterostructures [38,42].

This review attempts to provide the first comprehensive compilation of the current research on synthesis approaches, electronic structure, optical properties and photocatalytic activity of Cu_3_N nanostructures. Herein, we analyze the effect of synthesis conditions on the precipitation of Cu_3_N and the formation of secondary phases of Cu and Cu_2_O, with emphasis on shape and size control. In fact, synthesis conditions determine the shape, size and surface defects of the nanoparticle. These have a direct on influence the electronic structure and subsequently on the band gap, optical and photocatalytic properties of the materials. Furthermore, it summarizes the emerging applications of Cu_3_N in photocatalysis, and for the first time, to the best of our knowledge, a possible photocatalytic reaction mechanism of Cu_3_N is proposed. The outlook of the material in commercial applications and future research is described in the summary and outlook section.

## 2. Synthesis of Cu_3_N Nanomaterials

During the last decade, significant progress has been made in the nitride chemistry synthesis field. The main synthesis routes consist of solid–gas state synthesis, solvothermal, sol-gel, non-thermal plasma, electrospinning, electrodeposition, atomic layer deposition, chemical vapor deposition and laser ablation methods among others [43,44,45]. These new strategies for nanomaterial synthesis must consider the reaction conditions, along with the most relevant precursors in order to obtain reliable and reproducible properties of the synthesized materials. Compared to metal oxides, carbides, sulphides and oxynitrides, the synthesis of metal nitrides is relatively challenging due to the requirements of inert conditions during the growth process in order to suppress the formation of copper oxide. In addition, metal nitride nanostructures also tend to oxidize in ambient conditions, making their stability a challenging issue.

In particular, synthesis routes for Cu_3_N nanomaterials can be divided into chemical and physical. The chemical methods have been the focus of recent research for the synthesis of Cu_3_N nanoparticles, as they offer better control of the morphology and particle size distribution during the synthesis process. However, maintaining a controlled inert atmosphere in order to impede the formation of secondary phases (Cu, CuO and Cu_2_O) remains a concern. Alternatively, physical methods mostly focus on thin-film growth. In both methods, the growth of Cu_3_N nanomaterials requires different types of N sources, including metal-nitride, ammonia, alkylamines and nitrogen-based reagents. Solid–gas-state synthesis consists of Cu precursors reacting with nitrogen gas. On the other hand, solution-based syntheses are usually carried out in non-aqueous media in order to avoid oxidation. The synthesis methods for Cu_3_N nanostructures are summarized and discussed in detail below (Table 1).

### 2.1. Gas-state Synthesis and the Ammonia Source

**Figure 1:**
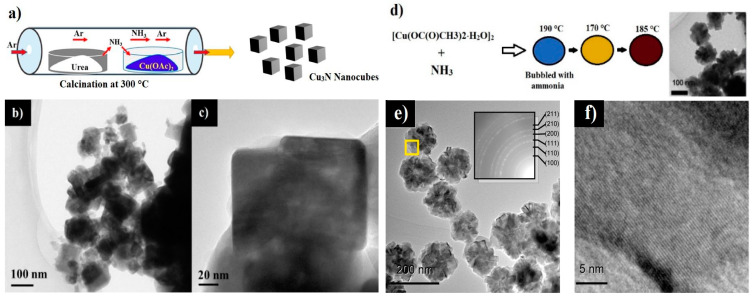
Schematic illustration of the synthesis and TEM micrographs of: Cu_3_N nanocubes using urea (**a**–**c**). Reprinted with permission from Ref [46], ACS publications, 2019. Schematic illustration of the synthesis of Cu_3_N nanoflowers using ammonia gas in long-chain alcohol solvent and corresponding TEM micrographs (**d**–**f**), adapted with permission from Ref [53], ACS publications, 2014.

In this method, the corresponding Cu-based metal precursor undergoes nitration under gas flow (i.e., NH_3_ or a mixture of N_2_:H_2_) to produce Cu_3_N nanostructures [72]. A variety of N-rich complexes, including hydrazine, urea, ammonium salt, carbon nitride and their mixtures, act as nitrogen sources in order to tailor the nanoparticle size and shape. Nevertheless, ammonia gas for ammonolysis has been widely used as it creates a reductive atmosphere and simultaneously acts as a powerful nitrogen source [73]. In ammonolysis, the reaction time, temperatures under 200 °C, heating and cooling rates, as well as the amount of ammonia are often adjusted in order to optimize the reaction conditions and the nanoparticle features. In addition, the properties of the produced nitride depend on the precursor and preparation conditions, such as heating rate and final temperature. For example, Panda et al. reported a method for the synthesis of Cu_3_N nanocubes by nitration of copper acetate at 300 °C inside a quartz tube, based on the in situ decomposition of urea leading to the release of ammonia, as shown in Figure 1a. Their TEM results show that the Cu_3_N nanoparticles are cube-shaped with particle sizes ranging from 60–100 nm presented in Figure 1b,c [46]. On the other hand, Nakamura et al. used cooper (II) acetate monohydrate in an alcoholic solution of 1-nonanol along with bubbling ammonia for 1 h at 190 °C to produce Cu_3_N nanoflowers (Figure 1d–f) [53]. In their study, the nanoparticles had granular shapes with a diameter of less than 200 nm. The differences in morphology and size arise from a combination of copper precursors, synthesis temperature and nitrogen sources. The utilization of urea as a nitrogen source, as opposed to NH_3,_ is very advantageous owing to facile handling and controllability of flux, implying that the risk of over-reduction of copper acetate to form metallic copper is mitigated [46]. The mechanism of nanoparticle formation was elucidated by Nakamura et al. and followed certain reaction steps: (1) formation of copper (II) amine complex, (2) reduction, by the long-chain alcohol, of Cu^2+^ to Cu^+^ and (3) the reaction between ammonia and Cu^+^ [53].

Multistep synthesis approaches have also been applied to the synthesis of Cu_3_N. These approaches are used to reduce copper oxide nanoparticles produced in the first step of the synthesis, followed by nitration. For example, Szczesny et al. synthesized Cu_3_N nanoparticles with diverse morphologies from oxygen-containing precursors using a two-step process that combines solvothermal and solid–gas ammonolysis stages [74]. In the first step, copper (II) chloride dihydrate was used as a precursor for the fabrication of copper (II) oxide and copper (II) hydroxide nanoarchitectures by solvothermal methods. In the second step, Cu_3_N was obtained through ammonolysis of Cu(OH)_2_ and CuO nanoparticles [74]. Deshmukh et al. synthesized Cu_3_N@SiO_2_ composites using a multistep approach [71]. First, CuO nanoparticles were synthesized within hollow mesoporous silica spheres by binding or adsorbing Cu^2+^ ions onto the surface of carbon spheres, followed by the formation of a mesoporous silica shell by a sol-gel method. Cu_3_N nanoparticles (size < 30 nm) were formed within silica spheres through the nitration of copper (II) oxide composite (CuO@SiO_2_) with ammonia gas at 300 °C for 10 h [71]. In the two-step approach, chemical nitration can only occur after the copper oxide is reduced to copper ions, which then react with ammonia to form Cu_3_N. During the synthesis, a change in the color of the solution from yellow to brown indicates the reaction of copper ions with ammonia leading to the precipitation of nitride nanoparticles [62]. Furthermore, Nakamura et al. suggested that for successful nitration, ammonia must come into contact with the copper ions at an optimum temperature as soon as the reduction of copper oxide starts [53].

The ammonolysis reactions provide a versatile route to synthesize Cu_3_N nanoparticles. Based on reports, ammonolysis is performed under specific conditions in solid-state chemistry in order to obtain a complete exclusion of oxygen and water during synthesis and handling. In addition, this approach can be optimized by varying the solid precursor, ammonia flow rate, heating and cooling rates, temperature and reaction time. However, the control of the nanoparticle shape and size in these processes remains a challenge. Moreover, this pathway involves the use of NH_3_, which is toxic and corrosive at high temperatures.

### 2.2. Solution-Based Synthesis

In addition to ammonia gas, nitrogen-based compounds can also be used as nitrogen sources for the synthesis of Cu_3_N nanostructures. In consequence, the use of non-aqueous solutions in the synthesis of metal nitrides has gained interest, as it suppresses oxidation and allows controlling the nanoparticle size and shape. The technique of non-aqueous synthesis requires water or hydrate-free solvents, as well as regulated temperatures and pressures in a controlled environment, as it relies primarily on the solubility and reactivity of precursors at relatively high temperatures and pressures [75]. Primary amines are commonly used as capping agents to synthesize metal nitrides and semiconductor nanomaterials. In general, solvothermal methods offer good control of the shape, size and crystallinity of nanostructured materials by controlling synthesis parameters, such as reaction time, temperature, precursor, surfactant and solvent, in order to vary the physical and chemical properties of the nanomaterials [76].

#### 2.2.1. Effect of Solvent

**Figure 2:**
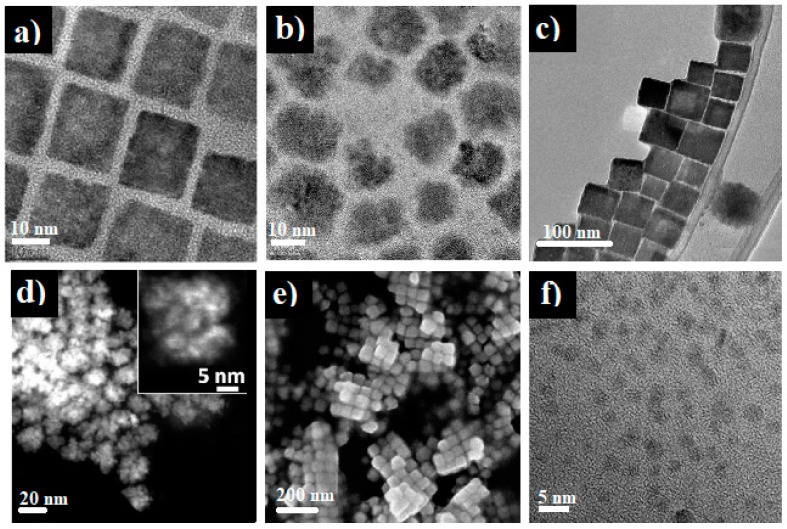
TEM micrographs of Cu_3_N nanostructures synthesized using different amines: (**a**) Cu_3_N nanocubes from Cu(NO_3_)_2_⋅3H_2_O in ODA. Reprinted with permission from Ref [49], RSC publications, 2011. (**b**) cubic-like Cu_3_N nanoparticles from Cu(NO_3_)_2_⋅3H_2_O in OAm. Reprinted with permission from Ref [38], ACS publications, 2019. (**c**) Cu_3_N nanocubes from Cu(NO_3_)_2_⋅3H_2_O in ODA. Reprinted with permission from Ref [48], Elsevier publications, 2019. (**d**) HAADF-STEM micrograph of Cu_3_N mesocrystals from PPC in ODA. Reprinted with permission from Ref [63], RSC publications, 2021. (**e**) FE-SEM of Cu_3_N cubic-like nanoparticles from Cu(NO_3_)_2_⋅3H_2_O in ODA. Reprinted with permission from Reprinted with permission from Ref [63] RSC publications, 2021. (**f**) ultrasmall nanoparticles from Cu(OMe)_2_) in BZA solvent. Reprinted with permission from Ref [54], RSC publications, 2015.

Presently, the thermal decomposition of copper salt-based precursors in long-chain amines or alcohols is one of the most popular synthesis techniques for Cu_3_N nanoparticles. It not only serves as a nitrogen source but can also performs simultaneous functions as a solvent, surface stabilizer and reducing reagent. Therefore, special attention is being paid to the synthesis of Cu_3_N nanoparticles using solvents, such as 1-octadecene (ODE) and amine sources, including 1-octadecylamine (ODA), hexadecylamine (HAD), oleylamine (OAm) and benzylamine (BZA) [33,38,47,49,54,63,77]. Synthesis of Cu_3_N nanocubes with tunable sizes was reported by Wu et al. through the decomposition of Cu(NO_3_)_2_ in ODE with different capping agents by a facile one-step process [47]. The nanoparticle size depended on the amine source used and ranged from 10 to 30 nm. In fact, the size was reduced significantly to 26 nm with ODA, to 18.6 nm with HAD and even further to 10.8 nm with OAm [47]. A similar synthesis approach for Cu_3_N nanocubes using ODA and ODE was also reported by Wang et al. [49]. They obtained Cu_3_N nanocubes (size = 15 nm) in Figure 2a after the thermal decomposition of Cu(NO_3_)_2_⋅3H_2_O at 240 °C in ODA solvent [49]. Similarly, Barman et al. reported the synthesis of Cu_3_N nanocubes with an average particle size of ~10 nm using a 1:1 volume ratio of ODE and OAm solvents [38]. The TEM image consists of smaller nanoparticles that coalesce into a nanocube, as visible in Figure 2b.

In addition, different solvents and reaction parameters influence the size and morphology of the nanoparticles. For example, Princ et al. synthesized two types of Cu_3_N nanoparticles with different morphologies using two different capping agents dissolved in ODE [63]. The report revealed that when OAm is used as a capping agent, spherical nanoparticles of 8 nm were produced (Figure 2d). In contrast, HAD produces cube-like nanoparticles of 50 nm (Figure 2e) [63]. Sithole et al., in two different reports, synthesized Cu_3_N nanoparticles and Cu_3_N nanocubes using amines as solvents [24,48]. Cu_3_N nanoparticles with an average particle size of 2.8 nm were synthesized using pyrrole-2-carbaldpropyliminato Cu(II) (PPC) as a single-source precursor with ODA [24]. On the other hand, Cu_3_N nanocubes with an average size of 41 nm, Figure 2c, were synthesized using Cu(NO_3_)_2_⋅3H_2_O at 240 °C in ODA [48]. Finally, Deshmukh et al. reported the synthesis of ultrasmall Cu_3_N nanoparticles of ~2 nm, Figure 2f, via a one-step reaction of copper (II) methoxide (Cu(OMe)_2_) precursor with BZA at lower temperatures for short reaction periods (e.g., 140 °C for 15 min) [54].

#### 2.2.2. Effect of Capping Agent

The above results suggest that the nanoparticle size and shape vary as a function of capping agents or surfactants. In fact, the long-chained amine surfactant plays an important role in shaping the nanoparticles at the nucleation and growth stages. They modify the decomposition routes of the copper nitride (II) reagent contrarily to other solvents or solid-state processes. According to Princ et al., the reasons for differences in the overall morphologies of nanoparticles using different capping agents may be related to the different mesocrystal subunits or the subsequent crystallographic orientations of subunits [63]. From the TEM images of Figure 2b–d, the coalescence of small spherical nanoparticles into nanocubes could also be another mechanism. The formation of nanocubes via such a mechanism appears to be more favorable in the case of long-chained amines. In fact, the subunits of nanocrystals are subject to various competitive forces, such as van der Waals, dipole–dipole and dissolution. Nonetheless, it is important to mention that so far, there is no report that elucidates the effect of the amine chain length on the size and morphology of the nanoparticles. Nevertheless, a complex combination of the synthesis environment, reaction time, temperature and amount of reagents influence the final shape and size of the nanoparticles.

#### 2.2.3. Effect of Reaction Time

**Figure 3:**
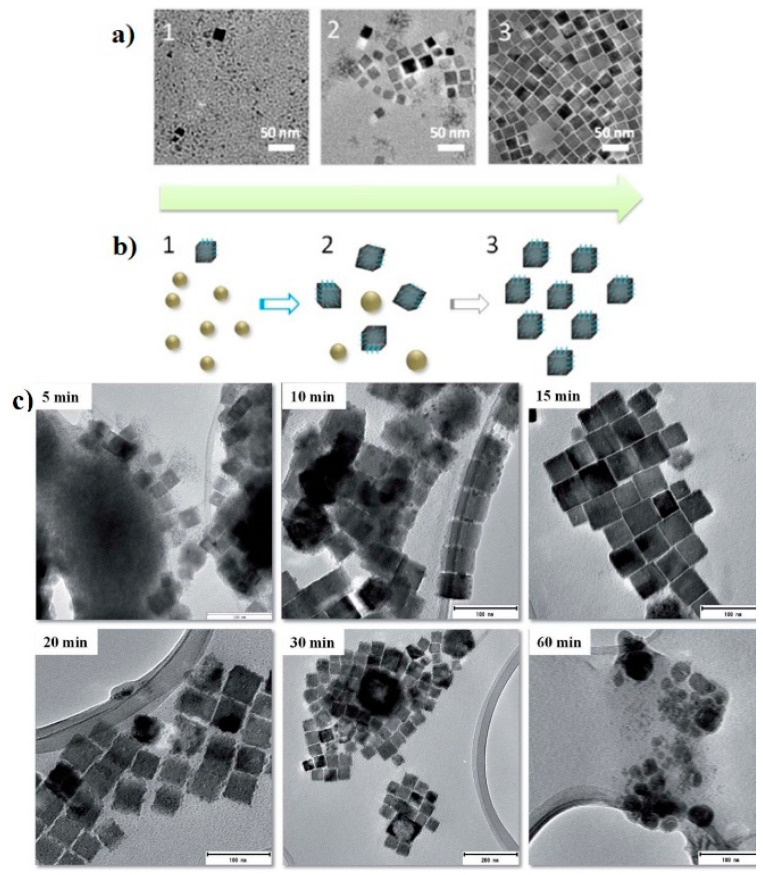
TEM micrographs of Cu_3_N nanocubes synthesized at different reaction times: (**a**,**b**) 2, 5, 10 min of reaction. Reprinted with permission from Ref [50], RSC publications, 2014. (**c**) 5, 10, 15, 20, 30 and 60 min of reaction time. Reprinted from Ref [33], Creative Commons agreement from RSC publications, 2019.

The reaction time related to the formation mechanism of Cu_3_N nanoparticles (nucleation, growth, aggregation and breakage) is a primary factor to be considered. It has been reported that the nanoparticle size, shape and stability are strongly dependent on the reaction time [78]. For example, Xi et al. reported the solvothermal synthesis of magnetic Cu_3_N nanocubes with high electrocatalytic reduction properties [50]. The cube-shaped Cu_3_N nanoparticles were synthesized by dissolving copper (II) nitrate in organic solvents of ODA and OAm at a high temperature of 240 °C. The detailed growth process of Cu_3_N nanocrystals is shown in Figure 3a,b, consisting of three different stages: (1) nucleation stage to form nanoparticles, (2) growth stage in which the nanoparticles adopt the cubic shape, owing to the surface tension exerted by the solvent and (3) molding stage in which nanocubes are formed [50]. In addition, Sithole et al. (shown in Figure 3c) reported a fourth step, i.e., the transformation of Cu_3_N into Cu nanoparticles [33], which is due to a spontaneous decomposition of Cu_3_N nanocubes depending on the reaction time. In fact, a cloud of nuclei with a few developed Cu_3_N nanocubes was obtained after 5 min, followed by well-defined nanocubes after 15 min. Finally, after 20 min of heating, degradation of the nanocubes occurs, and the copper nitride decomposes into metallic Cu within 60 min.

#### 2.2.4. Formation of Secondary Phases

Both routes have their share of advantages and drawbacks. One common disadvantage is the precipitation of secondary phases during Cu_3_N synthesis. For instance, it is reported that by prolonging the reaction time at a particular temperature, a partial phase transformation of Cu_3_N into Cu and Cu_2_O occurs [63]. Kieda et al. applied a spray pyrolysis technique using a copper–amine complex solution. However, obtaining a pure phase of Cu_3_N using only Cu_2_CO_3_(OH)_2_ precursor [79] is unlikely, as the co-precipitation of Cu, Cu_2_O and CuO secondary phases is inevitable. On the other hand, changing the molar ratio of the reactants (Cu(NO_3_)_2_⋅3H_2_O:ODA) results in the formation of Cu and Cu_2_O nanocrystals with or without Cu_3_N [33]. The formation of CuO is suppressed most likely due to a more reductive synthesis environment. Therefore, the development of a scalable, reliable and reproducible method for the synthesis of high-quality Cu_3_N nanostructures with a high density of active sites is the need of the hour. Additionally, the degassing process, which removes water molecules from the solution, is an important step in suppressing the formation of secondary phases. The most commonly used gases are nitrogen or argon, which eliminate water molecules from the solution while heating the solution between 100 °C to 150 °C. Wet chemical routes tend to be more reliable and reproducible in controlling nanoparticle shape and size compared to gas-state methods, thus making them viable for larger-scale production. Major limitations in upscaling these methods include high reaction temperatures and the use of surfactants, which increase their costs and complexity.

### 2.3. Thin Film Deposition Techniques

Several studies have focused on the growth of Cu_3_N thin films. A number of techniques, such as DC and RF reactive magnetron sputtering, thermal evaporation, pulse laser deposition (PLD) and CVD, are commonly used to grow Cu_3_N thin films. These deposition techniques produce nitride thin films with controlled stoichiometry, thickness and composition [39]. In fact, the efficient thin film deposition of Cu_3_N has mainly been achieved via physical methods for applications in optical data storage [80], solar energy conversion [15], BioMEMs [81] and photovoltaics [82]. In general, physical methods are useful for growing single-phase thin films but have the drawback of producing only a limited variety of compounds [76]. Furthermore, the doping of Cu_3_N with other compounds has marked a breakthrough in nitride thin film research. In that regard, thin-film deposition techniques provide a simple pathway to introduce foreign atoms while retaining the Cu_3_N lattice stability.

**Figure 4:**
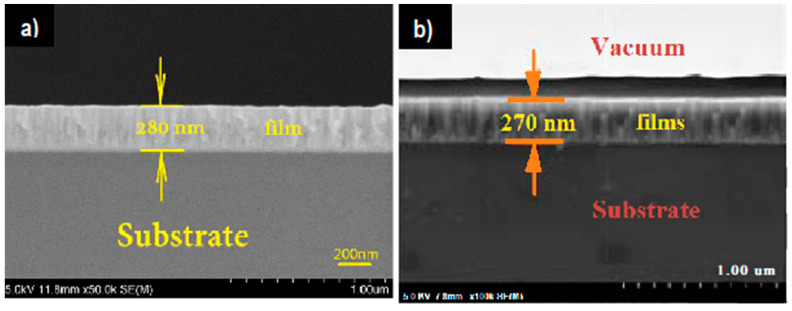
Cross-section view of thin films: (**a**) Cu_3_N:Pb thin film. Reprinted with permission from Ref [30], Elsevier publications, 2019. (**b**) Cu_3_N:Ag thin film. Reprinted with permission from Ref [69], IOP publishing, 2018.

Most of the previous work on Cu_3_N nanostructures has been on thin film deposition using magnetron sputtering. The method is often used to produce transition metal nitride thin film electrodes, which exhibit excellent adhesion, controllable composition and thickness of nanostructured films with excellent electrochemical properties [83]. During the reactive sputtering of Cu_3_N, a reductive gas such as N_2_ or Ar reacts with a Cu metal or a Cu_3_N target, sputtering off the compound layer. Then, the sputtered Cu and N species are deposited on substrates forming Cu_3_N compound layers [68]. For instance, Jiang et al. deposited Cu_3_N:Pb thin films using different Pb concentrations on monocrystalline silicon through DC and RF reactive magnetron sputtering [30]. In fact, Pb doping leads to a high adhesion of the films to the substrate, as it promotes nucleation in the early stages of thin-film growth [30]. Figure 4a shows the cross-sectional view of dense and well-adhered films. Similarly, Xiao et al. investigated the growth of Cu_3_N:Ag thin films using RF and DC magnetron sputtering (Figure 4b) [69]. In this research, Cu_3_N:Ag thin films were prepared with variable sputtering power of Ag. They observed that an increase in the Ag content brings about an increase in the grain size. Additionally, the optical band gap of the films also increases owing to the plasmonic-induced Burstein–Moss effect leading to an increase in the number of carriers in the conduction band of Cu_3_N, which in turn displaces the Fermi level towards the conduction band [69]. In both Ag and Pb doped thin films, the growth mode is columnar, typical of the sputtering deposition process. However, the roughness of the Pb doped films is higher than the Ag-doped films, most probably due to the Pb atom being slightly larger than the Ag atom. Since the lattice mismatch for Pb doped films is higher, the lattice relaxation process enhances the thin film roughness. Similarly, nanocomposites of Cu_3_N tend to have enhanced optical properties. For example, TiO_2_-Cu_3_N nanocomposites manifest an improved bandgap compared to Cu_3_N alone. Zhu et al. also reported the growth of TiO_2_-Cu_3_N and Cu_3_N-MoS_2_ nanocomposite films by magnetron sputtering that demonstrate enhanced photocatalytic properties [35,84]. Both TiO_2_ and MoS_2_ are n-type semiconductors that, when combined with p-type Cu_3_N, create p-n junctions. For catalytic activity, large specific surfaces of active materials are required. Moreover, when the roughness increases, the number of active sites on the surface also augments, which leads to an enhancement of photocatalytic activity [35,84]. Industrially viable thin film growth techniques, such as sputtering, allow thickness control with the possibility of depositing on large surface areas in order to obtain a high specific surface. The deposition parameters of Cu_3_N thin films include sputtering power, N_2_ pressure r (r = N_2_/[N_2_^+^ Ar]), substrate temperature and deposition pressure, which have a significant influence on Cu_3_N thin film growth and their properties. Additionally, deposition parameters also affect the crystal orientation and grain size of Cu_3_N thin films [65,83]. 

**Figure 5:**
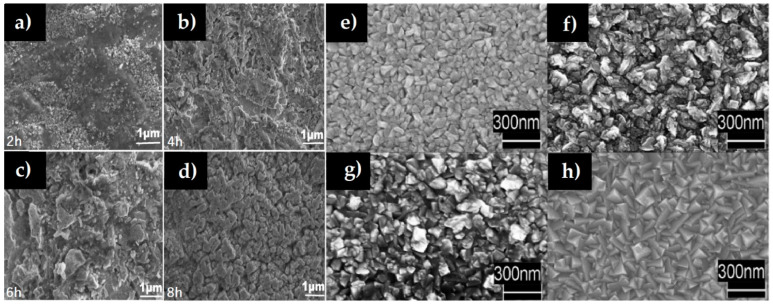
(**a**–**d**) SEM images of Cu_3_N thin films grown by thermal evaporation technique at different reaction times. Reprinted with permission from Ref [67], Elsevier publications, 2021. (**e**–**h**) SEM images of Cu_3_N films with different Ni metal content: (**e**) pure Cu_3_N, (**f**) 21% Ni, (**g**) 46% Ni and (**h**) 76% Ni. Reprinted with permission from Ref [29], Elsevier publications, 2018.

Cu_3_N thin films can also be deposited by thermal evaporation using nitrogen as a gas source. In this method, the Cu powder is evaporated onto a glass substrate and then exposed to N_2_ gas in order to fabricate Cu_3_N thin films. Ali et al. thermally evaporated Cu powder on a glass substrate in a horizontal glass tube furnace at 1000 °C in a high vacuum for 25 min [67]. Then, the temperature of the tube furnace was set to 300 °C, and an N_2_ gas flow rate of 100 sccm was maintained for different reaction times. Figure 5a–d show the SEM images of Cu_3_N thin films deposited for a duration of 2, 4, 6 and 8 h under nitrogen gas flow at 300 °C. When the growth time is prolonged, an increase in the grain size is observed [67]. Cu_3_N thin films with a rough surface were deposited by Lindahl et al. using a gas pulsed CVD [29], which is a gas phase chemical method by which thin films are grown on a suitable substrate through precursor decomposition at elevated temperatures. In that case, Cu-Ni-N thin films were prepared using CVD processes with intermittent NH_3_ gas flow. Ni-doped Cu_3_N can be described as a solid solution of Ni in Cu_3_N formed in a pseudo-binary system composed of the two metastable phases Cu_3_N and Ni_3_N (Figure 5b). According to Lindahl et al., the texture of the deposited films changes with the increase of Ni content, which was attributed to changes in the deposition mechanism through the probable occupancy of the interstitial sites of Cu_3_N in the crystal structure [29].

## 3. Electronic Structure and Band Gap

**Figure 6:**
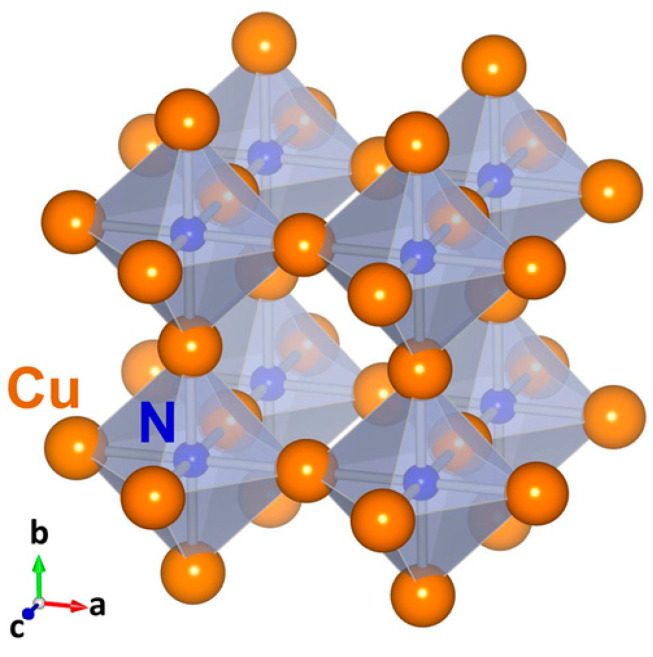
Schematic view of the anti-ReO_3_ crystal structure of Cu_3_N. Reprinted with permission from Ref [85], Wiley publications, 2018.

Cu_3_N has a primitive open anti-rhenium trioxide cubic structure (space group Pm3m, lattice constant a = 3.82 Å), presented in Figure 6 [85]. In the structure, N atoms occupy the cube corners of the unitary cell in (0, 0, 0) and Cu atoms are located between two consecutive nitrogen atoms in (½,0,0), (0, ½, 0) and (0, 0, ½) [26,51,86,87]. Each N atom in the cubic structure of Cu_3_N is shared by eight cells, while each Cu atom is shared by four cells. In pure Cu_3_N, the face centers and the body of the cube are empty. These void interstitial sites are, therefore, available to host Cu, N or other foreign atoms under certain conditions [88]. However, the insertion of additional metallic elements in the body of the Cu_3_N lattice alters the chemical interactions between Cu and N, thereby modifying or changing the electrical and optical properties. Experimental and computational studies reveal that metal-doped Cu_3_N adopts metallic characteristics due to the reduction of the metallic inclusions (M^0^) [88]. For instance, Hahn et al. demonstrated that Cu_3_N is a semiconductor, while Cu_3_N-Pd exhibits semi-metallic conductivity due to Pd atoms causing modifications in the energy bands [89]. Additionally, Cu_3_N:Pd presents higher mass activities and stability in comparison with commercial Pd alone. In addition, Moreno et al. established that the addition of an extra Cu atom into the body of the unitary cell of Cu_3_N endows it with metallic properties [90]. In turn, the unit cell of Cu_3_N expands, owing to the progressive insertion of Cu atoms. Furthermore, their computational studies show that the lattice parameter of Cu_3_N-Cu expands by 0.06 Å. Moreno et al. inferred that Cu_3_N with lattice constants higher than 3.868 Å behave as conductors [90]. Finally, these types of doped-compounds, i.e., Cu_3_M_x_N, present an anti-perovskite structure, owing to the metal dopant occupation of (½, ½, ½), i.e., the body-center position [88]. Anti-perovskite structures consist of interchanged A and B site atoms. They are defect tolerant, present excellent ionic conductivities and are ideal candidates for electrodes of batteries [91]. This suggests that they possess interesting redox capabilities, enabling their application as photocatalysts.

Furthermore, the crystal chemistry of transition metal nitrides is largely dictated by the bond coordination of N and M atoms. In these compounds, the M atoms are bonded with nitrogen via covalent or ionic bonds. According to Rao et al., the addition of N atoms to a transition metal converts the metal–metal bond to a covalent bond or a mixture of covalent–metal bonds [44]. Moreover, the addition of N increases the bond length and, consequently, the lattice parameters, resulting in a contraction of the metal d-band, further suggesting that bonds in metal nitrides are ionic. This d-band contraction causes an increased density of states (DOS) near the fermi level, giving rise to novel catalytic properties, which are different from the parent metal but rather similar to noble metals [14]. Moreover, recent studies suggest that both covalent and ionic bonds connect Cu and N atoms in undoped Cu_3_N [30]. On the other hand, for M-doped Cu_3_N, the bond coordination has a covalent character as the interactions between the inserted M and Cu atoms dominate [42,92].

Undoped Cu_3_N tends to be insulating due to defect states created by metallic and non-metallic vacancies of Cu and N, respectively [93]. However, due to the varying nature of the vacancies giving rise to shallow defect states, the bandgap of Cu_3_N shows variations and ranges from 0.2 to 2.0 eV. Another study demonstrated that doping with iodine gives rise to states that are located very close to the band edges and therefore resulting in the absence of band gap states [94]. Furthermore, Cu_3_N being a p-type semiconductor, displays a strong hybridization of the Cu 3d and N 2p orbitals close to the valence band owing to their anti-bonding states, which causes their high tolerance to defects. Theoretical calculations confirm that Cu 3d electrons are a major contributor to the DOS and have an important influence on the Cu_3_N band gap.

Since nanostructured defect-tolerant transition metal nitrides possess semiconducting properties with band gaps corresponding to visible wavelengths, they are therefore considered suitable materials for photocatalysis and photovoltaic applications [39,95]. In the literature, there are several discrepancies in the band gaps of Cu_3_N arising from both theoretical and experimental studies. Theoretical calculations predict an indirect band gap ranging from 0.23 to 1.0 eV depending on the calculation method [58,88]. Density Functional Theory (DFT) tends to underestimate the band gap mainly due to self-interaction errors and changes in potential upon changing the number of electrons in the conventional framework of DFT [96]. Co-workers also calculated the band structure and DOS before and after the addition of a foreign atom into the cubic structure of Cu_3_N. In fact, the electronic structure changes in the presence of foreign atoms, accompanied by an increase in the Fermi energy, along with a consequential change in the band gap. For instance, computational calculations reveal that pure Cu_3_N is a semiconductor with a small indirect band gap equal to 0.38 eV. In contrast, for M-doped Cu_3_N (M = Sc, Ti, V, Cr, Mn, Fe, Co and Ni), the band gap becomes negligible, giving rise to the metallic character of Cu_3_N [42]. Other studies confirm a semiconducting behavior but suggest that pure Cu_3_N presents an indirect band gap of 0.32 eV and direct gaps of 1.09 and 0.87 eV, whereas for Cu_3_N-N rich compounds, a partially filled spin-resolved narrow band of new electronic bandgap states at the Fermi energy is observed [97]. This new band modifies the optical properties of Cu_3_N and makes the material susceptible to infrared absorption.

On the other hand, experimental results ascertain both direct and indirect band gaps ranging from 1.17 to 2.38 eV that depend on the experimental conditions and N content. Table 1 summarize the synthesis parameters and band gaps of Cu_3_N and Cu_3_N-M nanostructures. Band gaps determined experimentally vary, for example, between 1.91 to 2.15 eV for Cu_3_N thin films deposited by sputtering, whereas a maximum band gap of 2.92 eV was reported for nanoparticles of Cu_3_N [52]. Furthermore, band gaps of 1.04 eV (indirect) and 1.5 eV (direct) were obtained from cyclic voltammetry (CV) measurements on Cu_3_N nanocubes based on onset Redox potentials [47]. In general, the experimental band gap depends on the synthesis conditions, which in turn determines the morphology and chemical composition of the nanomaterial.

## 4. Photocatalytic Activity of Cu_3_N Nanoparticles

Photocatalysis is a redox process involving three main steps: (i) the generation of electron-hole pairs (e^−^/h^+^) by photoexcitation, (ii) transportation of the excitons to the semiconductor surface and (iii) the utilization of charge for surface oxidation–reduction reactions [39,41]. The total efficiency in these processes is strongly determined by the properties of the semiconductor photocatalyst, such as the electronic structure, band gap, surface properties along with kinetic and thermodynamic processes [98]. An ideal photocatalytic material should primarily have a bandgap that is optimal for absorbing in the entire range of the solar spectrum in order to dissociate water molecules while retaining its stability during the reaction process. In addition, it must be cost-effective, easy to process, readily available and non-toxic. Moreover, in order to efficiently harvest visible light and accelerate reactions, the band gap should preferably be under 3.2 eV. However, since redox reactions require a certain amount of energy, narrow band gap semiconductors may therefore not be the most suitable for catalytic reactions. In addition, the probability of excitonic recombination increases for lower bandgaps. Therefore, an optimum balance between the band gap and recombination lifetime is required. One method for efficient charge separation is the combination of plasmonic nanoparticles with photocatalytic semiconductors.

Current reports demonstrate a keen interest in the photocatalytic activity of metal nitride nanomaterials. Their metal-like properties, such as conductivity, optical band gap and visible light activation, are beneficial for highly efficient separation and delivery of photogenerated carriers. Unlike traditional semiconductors (oxides, sulfides and carbides), metal nitrides can lower the overpotential or activation energy for photocatalytic reactions on the surface of semiconductors. They also provide additional active sites and promote electron-hole separation at the interface of the co-catalyst [39,99]. Furthermore, their low-cost production, high thermal stability and tolerance against acids and bases are advantages in photocatalysis [39]. Inspired by these properties, the design and construction of semiconductor-based metal-nitride nanomaterials have been carried out and applied to photocatalytic processes for water splitting, organic dye degradation, CO_2_ reduction and decomposition. For example, CoN, [100] Ta_2_N, [100] Ta_3_N_5_, [101], Ni_3_N, [102,103] and InGaN [104] have been tested as potential semiconductors for water splitting. In photocatalytic water splitting reactions, the photo-induced process enables the production of H_2_ and O_2_ [44,105]. Additionally, metal nitrides are sturdy with good refractory properties at elevated temperatures of 2000 °C owing to their metallic character. The latter also dotes them with properties similar to plasmonic metal nanoparticles. However, compared to noble metal nanoparticles, nitrides display plasmonic wavelengths in the visible and infrared regions. For ex., TiN has been used as plasmonic interconnectors, further suggesting that nitrides can replace noble metals in such applications. Nitrides also display tunable optical responses and improved light-harvesting, equivalent to noble metals. They can, in general, significantly enhance the local electromagnetic field when the incident light interacts with the surface plasmons [44,106]. In other words, localized surface plasmon resonance (LSPR) enhances the electric field, facilitating light absorption and charge transfer processes of semiconductors [38,107]. In such cases, the generated excitons have higher energy than the Fermi level of the photocatalyst, which is ideal for driving photocatalytic reactions [108]. For example, ZrN [109], HfN [110] and TiN [111] are reported as plasmonic metal nitride nanostructures. However, Cu_3_N has not yet been specifically reported as a plasmonic nanomaterial. Nevertheless, synthesis routes tend to produce secondary phases of Cu metal, which is a known plasmonic material that enhances the overall catalytic performance of the primary phase [33]. In all cases, metal nitrides need to be combined with a metal or themselves possess a metallic character in order to display surface plasmon resonance. This is likely if they are synthesized with an excess of Cu in the case of Cu_3_N. In soft chemical synthesis, excess Cu could favor the co-precipitation of Cu metal and simultaneously incorporate Cu in the Cu_3_N lattice. For thin-film synthesis, the Cu metal phase separation can be suppressed by controlling the growth conditions in order to ensure the incorporation of Cu in the Cu_3_N lattice.

**Figure 7:**
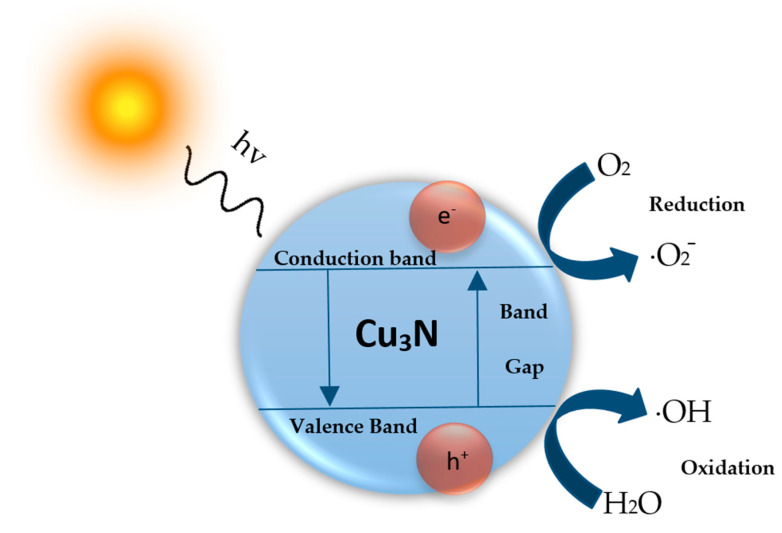
Schematic representation of photocatalytic mechanism for Cu_3_N photocatalyst.

With further regard to Cu_3_N-based nanostructures, several studies have reported photocatalytic degradation of dyes. A schematic representation of the Cu_3_N-based photocatalytic process is represented in Figure 7. The process starts when Cu_3_N absorbs energy equivalent to its bandgap, whereupon generating a wide range of electron-hole pairs (e^-^-h^+^) under the photoelectric effect. The e^-^-h^+^ pairs migrate to different positions on the surface of the Cu_3_N under the action of the generated electric field. The electrons on the surface of Cu_3_N are then transferred to the adsorbed molecular oxygen, generating reactive oxygen species such as superoxides (•O_2_−). The holes react with water and hydroxyl ions to generate hydroxyl radicals (•OH) that can oxidize organic matter adsorbed on the surface of Cu_3_N. The oxidizing ability of •OH radicals is the most potent among reactive oxygen species in aqueous media. It is capable of oxidizing most of the inorganic contaminants, as well as organic matter in water and degrading them to smaller inorganic molecules such as carbon dioxide, water and other harmless substances. The possible chemical reactions of the photocatalytic activity of Cu_3_N are as follows:

Cu_3_N + *hv* → e^−^ + h^+^

h^+^ + H_2_O → •OH + H^+^ (1)

e^−^ + O^2^ → O_2_^−^

OH + H^+^ *dye* →…→ CO_2_ + H_2_O.

In general, Cu_3_N demonstrates excellent electrochemical performance. Both photo- and electrocatalysis have the same mechanism of charge creation. The difference lies in the type of activation energy provided to create these charges. Therefore, electrochemistry can elucidate the redox reactions of Cu_3_N in order to evaluate its potential for hydrogen and oxygen production. Sajeev et al. evaluated the potential of Cu_3_N in hydrogen evolution reaction (HER), where they claim a lower overpotential than its oxide counterpart Cu_2_O [112]. In general, the shift from oxides to nitrides for photo- or electrocatalysis has been facilitated owing to the higher potential of N 2p than O 2p orbitals. This signifies that metal nitrides have a lower bandgap than metal oxides, whereby they are more adapted to photocatalysis. They can therefore be also employed as working photoelectrodes for photocatalytic water splitting. Nevertheless, Cu_3_N tends to be unstable in aqueous media and high and low pH conditions due to its tendency to oxidize. However, these concerns are surmounted by synthesizing nanocomposites of Cu_3_N [98], which are particularly useful for dye degradation in aqueous media. Cu_3_N is also used as an electrocatalyst in gas-phase reactions for the production of hydrocarbons via CO_2_ reduction reactions [113]. Presently, there are no accounts of photoelectrocatalytic production of hydrocarbons with Cu_3_N or its nanocomposites, even though the potential exists.

**Figure 8:**
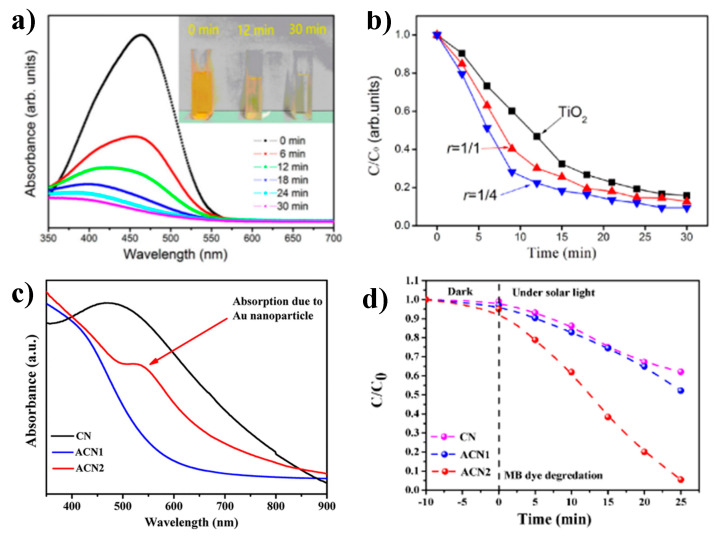
(**a**) Methyl orange degradation by UV-Vis analysis using Cu_3_N thin films. Reprinted with permission from Ref [65], Creative Commons agreement from MDPI, 2020. (**b**) Degradation of methyl orange by TiO_2_ films and TiO_2_@Cu_3_N composite films prepared by different gas flow radio (r = [N_2_]/[Ar + N_2_]), at r = 1/4 the degradation rate of MO is 99.2%. Reprinted with permission from Ref [84], Creative Commons agreement from Elsevier publications, 2021. (**c**) UV-Vis spectra of Cu_3_N nanocubes and Au-decorated Cu_3_N nanocubes (ACN1 Au nanoparticles is ∼5 nm and ACN2 Au nanoparticles is ∼10 nm) and (**d**) Solar light driven photo degradation of MB using Au-decorated Cu_3_N nanocubes. Reprinted with permission from Ref [38], ACS publications, 2019.

In recent years, several studies of Cu_3_N nanomaterials for photocatalytic applications have been reported (Table 2). Jiang et al. reported the photocatalytic degradation of methyl orange (MO) using Cu_3_N thin films with an optical band gap of 2.0 eV [65]. In this work, a MO solution of 20 mg/L in 50 mL water was used as the target degradation product and Cu_3_N thin films with dimensions of 2.5 cm × 1.0 cm × 120 nm as a photocatalyst under a high-pressure mercury lamp (500 W). A degradation yield of 95.5% in 30 min was achieved (Figure 8a). They infer that the effective photogenerated electron-hole pairs mainly originate from Cu vacancies and interstitials in the film [65]. Although the Cu_3_N thin-film alone presents excellent photocatalytic properties for degrading dyes, it has a few shortcomings. Therefore, Cu_3_N nanostructures are combined with other semiconductors. For instance, Zhu et al. demonstrated that the degradation of MO using TiO_2_@Cu_3_N thin films (effective band gap = 3.0 eV) is higher than in pure TiO_2_ thin films, as seen in Figure 8b [35]. A degradation rate of 99.2% of MO solution with an initial concentration of 20 g/mL using a 500 W mercury lamp for 30 min was obtained [84]. Other studies using Cu_3_N@MoS_2_ thin films (band gap = 2.05 eV) as photocatalysts obtained a 98.3% degradation rate of MO with an initial concentration of 10 mg/mL in 30 min. In this case, controlled quantities of MoS_2_ reduce the overall band gap of the nanocomposite to values lower than the band gap of Cu_3_N [35]. On the other hand, during the past few decades, researchers have also studied the photocatalytic activity of Cu_3_N nanoparticles in aqueous media. Sithole et al. reported the photocatalytic degradation of methylene blue (MB) and MO using Cu_3_N nanocubes with a band gap of 2.41 eV [48]. In that study, 0.1 g of Cu_3_N nanocubes were added into the solution of MB and MO, with an initial concentration of 20 ppm under a solar simulator (AM 1.5 G 100 mW/cm^2^). Degradation efficiencies of 89% of MO after 180 min and 61% of MB after 240 min were obtained. They argue that the difference in degradation efficiencies was due to differences in the chemical structure of the dyes. They affirm that catalytic reactions are more efficient in anionic than cationic dyes. Similarly, Barman et al. evaluated the photocatalytic degradation of MB and MO using Cu_3_N-Au heterojunction as a photocatalyst (Figure 8c) [38]. The Au-Cu_3_N heterostructures ACN1 (Au nanoparticle size is ∼5 nm) and ACN2 (Au nanoparticle size is ∼10 nm) exhibit enhanced photocatalytic activity in comparison with pure Cu_3_N nanocubes, mostly due to the LSPR effect of Au nanoparticles that enhances charge carrier separation, Figure 8c. In addition, the ACN2 sample with an Au particle size ∼10 nm presents much better photocatalytic behavior in MB under solar radiation. In this case, the enhancement of the photocatalytic activity requires coupling between the conduction band of Cu_3_N and the Fermi level of plasmonic metals. Subsequently, charge carriers in semiconductors undergo plasmon-induced resonance energy transfer [38], which can improve the photocatalytic activity towards dye degradation.

Since photocatalysis is a surface phenomenon, the size of the nanocatalyst is a crucial parameter in evaluating the efficiency of its photocatalytic activity [114]. The decreasing size of the nanoparticle increases its specific surface, i.e., the number of active sites needed for the production of ROS on photoexcitation. Active sites should be able to chemisorb the dye molecule and simultaneously transfer the photogenerated charge carriers to them. These active sites could be defects such as Cu or N vacancies at the surface of the nanoparticle [112]. These surface defects are dominant in nanoparticles and augment with decreasing size of the nanoparticle. In addition, the morphology of the nanoparticles also plays an important role. Different morphologies such as rod, flower and cube tend to have different photocatalytic efficiencies. For example, cubic, octahedral and spherical platinum (Pt) nanoparticles present different photocatalytic activities under visible light [115]. Even though Cu_3_N nanoparticles exhibit high photocatalytic efficiencies of 80%, combining them with plasmonic nanoparticles of Au further increases these efficiencies to 90%. Compared to nanoparticles, thin films of Cu_3_N demonstrate extremely high efficiencies of 95%, most likely due to better control of the stoichiometry during thin film growth. Furthermore, the formation of secondary phases is easier to suppress during thin film growth by varying the growth parameters. In contrast, chemical routes for nanoparticle synthesis inadvertently generate secondary phases of Cu and Cu_2_O that influence the overall photocatalytic activity of Cu_3_N. Moreover, nanocomposites of Cu_3_N consisting of TiO_2_ and MoS_2_ show a higher photocatalytic dye degradation efficiency [35,84]. In such heterostructures, the Fermi level alignment reduces the overall bandgap of the material, making the nanocomposite more susceptible to visible light absorption and thereby increasing the absorption cross-section.

Cu_3_N-based hybrid nanomaterials are considered stable and can be reused in environmental remediation applications. Other than photocatalytic dye degradation in wastewaters, other environmental remediation applications of Cu_3_N are also available. Li et al. synthesized 3D Hierarchical Ni_4_N/Cu_3_N nanotube arrays for the electrolysis of urea, which is a common effluent in wastewater [116]. Furthermore, Lee et al. demonstrate that the catalytic activity of copper-based nanomaterials shows high stability and reusability. They examined the reuse of the Cu_3_N/Fe_3_N@SiO_2_ magnetic catalyst. The recycled catalysts were separated by a magnet and presented high stability and identical yield to those of the virgin catalyst after five cycles [92]. Yin et al. also support the stability and reusability of Cu_3_N nanoparticles. They report a new perovskite-type copper (I) nitride (Cu_3_N) nanocubes catalyst for selective carbon dioxide reduction reaction (CO_2_RR), and they demonstrate the reproducibility of their results after several cycles [113].

## 5. Other Applications of Cu_3_N

Not only does Cu_3_N exhibit high photocatalytic activity, but it also has appreciable catalytic activity for a range of important reactions. For example, Cu_3_N is considered a novel catalyst in the fields of sustainability, energy and the environment. Studies of the electrocatalytic activity of oxygen and hydrogen evolution reactions (OER and HER, respectively) and in the oxygen reduction reaction (ORR) have already shown that Cu_3_N holds considerable promise for energy production systems [47,50,113]. Furthermore, recent reports also indicate that Cu_3_N can be very effectively used as a catalyst in the CO_2_RR. Yin and co-workers demonstrated that Cu_3_N nanocubes were stable and selective to ethylene production at −1.6 V, with a Faradaic efficiency of 60% [113]. The electrocatalytic activity of Cu_3_N is, therefore, also suitable for electrodes of Li- and Na-batteries, owing to lithium-copper nitride reversible processes for a large number of cycles at elevated temperatures [55,117,118]. The material displays high optical absorption and has therefore been applied to photovoltaics [119]. In the electronics industry, Cu_3_N has successfully shown optical data storage capabilities when coupled with an Al_2_O_3_ protective layer [74]. The capacity to transform Cu_3_N under laser pulses of different wavelengths to Cu is the basis of optical data storage, measured in terms of changes in the reflectivity of the nanomaterial [80]. However, the thermal stability of Cu_3_N under laser irradiation is not suitable for spintronic applications. Therefore, FeN was combined with Cu_3_N, and the nanocomposite subsequently demonstrated potential for spintronic applications [120]. Nevertheless, the ability of Cu_3_N to decompose is an asset for other applications, such as Si-based large-scale integrated circuits. These Cu-metal lines on Si wafers offer higher signal speed than traditionally used Al-metal lines [121]. Furthermore, patterning substrates by exploiting the phase separation of Cu from Cu_3_N allows applying the plasmonic effect of the segregated Cu nanoparticles to Surface-Enhanced Raman Scattering (SERS) applications [122,123]. In fact, SERS is already being applied in several biomedical fields, such as bio-imaging, cancer diagnostics and plasmonic sensors [124,125]. For antimicrobial applications, Cu is considered a potent nanomaterial. However, in high doses, Cu presents cytotoxicity also. Therefore, careful control of Cu quantities is required. Furthermore, the antimicrobial properties of NiTi were enhanced when Cu_3_N-Cu catalyst was deposited on its surface, owing to a micro-galvanic effect [126]. Antibacterial activity against two strains of bacteria, i.e., *E. coli* and *S. aureus* were evaluated. In fact, the HER of the Cu_3_N-Cu catalyst induces oxidative stress on the bacteria through the production of ROS.

## 6. Summary and Outlook

The global market for nanomaterials has grown considerably in the last few years. Recent reports suggest an increase in the production of nanomaterials with a compound annual growth rate (CAGR) of 14.1% between 2021 and 2028. Among the various types of nanomaterials, nanoparticles are likely to account for the largest market share. Recent promising results on their applications in the health, energy, electronics and water treatment sectors have been a major driving force towards their commercialization. However, the synthesis of nanoparticles with specific properties for different applications is one of the greatest challenges for researchers, especially in water treatment applications, including photocatalysis. There are several photocatalytic nanomaterials in the market, with TiO_2_ and ZnO being the most effective. However, these nanomaterials suffer from certain drawbacks, such as inefficient absorption of visible light, large band gaps, the low adsorption capacity of hydrophobic contaminants, non-uniform distribution in aqueous suspension and reclamation methods after treatment. In addition, recent reports mention that there are regulatory restrictions in Europe on the use of TiO_2_ due to concerns about its genotoxicity [127]. Therefore, novel and industrially viable photocatalysts are necessary in order to surpass these limitations.

Environmental pollution, including water contamination, is a global concern that deteriorates ecosystems and negatively affects the climate. Today, climate change is the focus of the hour. Wastewater effluents generated daily by chemical, electroplating and metallurgical industries contain various organic dyes, e.g., methyl orange, rhodamine B and methylene blue. These pollutants cause damage to water resources, in addition to causing harm to humans and aquatic life owing to their toxicity and carcinogenicity. In the literature, there are strategies to deal with dye-contaminated waters involving physical, chemical and biological methods [128]. However, their disadvantages, such as low efficiency and incomplete contaminant removal, need to be further addressed. In this regard, photodegradation using nanoparticles has gained enormous popularity in recent years. Photobleaching of dyes is considered a favorable technology for industrial wastewater treatment due to its environmental friendliness, low cost and absence of secondary pollution. Therefore, photocatalysis research has become a niche topic in water treatment.

In the present scenario, metal nitrides appear as a versatile class of materials of growing interest. Cu_3_N specifically has attracted attention as an inexpensive, non-toxic material with potential applications in solar cells, high-density optical data storage, lithium-ion batteries, photocatalysis, electrocatalysis and photoelectrocatalysis. Chemical synthesis routes lead to the formation of nanocrystals with characteristics that can be controlled by varying the synthesis parameters. In fact, the size and shape of the nanoparticles are sensitive to the reaction time, temperature and chemical reagents. Although there are a good number of reports on the synthesis of pure phase Cu_3_N, there is still progress to be made, as the routes are often multistep, may involve the use of hazardous gases such as ammonia and provoke the co-precipitation of different phases, such as Cu_2_O and Cu during the synthesis process. Cu_2_O is already a well-established photocatalyst, and Cu has shown a synergistic effect on the latter. Therefore, Cu_3_N could be an excellent co-catalyst as it widens the visible absorption range of Cu_2_O. The large discrepancy in the bandgap of Cu_3_N depends on the synthesis conditions, including doping, which can be considered an advantage when tuning the bandgap of the material, especially when used as a co-catalyst with Cu_2_O. Similarly, thin films based on Cu_3_N have been extensively studied. Sputtering deposition of Cu_3_N thin films being industrially viable, can be used to coat large surface areas. Substrate dimensions on industrial sputtering lines can be as large as 1 m × 1.5 m. Since photocatalysis is a surface phenomenon, a large surface area coverage of Cu_3_N as obtained by such deposition techniques is necessary to ensure efficient photoabsorption and enhanced surface reactions. Therefore, Cu_3_N nanostructures could be good candidates as catalysts or co-catalysts in the photodegradation of polluted waters.

Cu_3_N nanomaterials present enormous potential for applications in several fields. However, the low stability of Cu_3_N remains a shortcoming. The synthesis of pure Cu_3_N without the presence of secondary phases will be the target for the coming decade. The field of application of Cu_3_N nanomaterials and nanocomposites is very broad and includes energy and environmental applications, such as catalysis, photocatalysis and electrocatalysis (OER, HER and ORR), as mentioned in this review. Furthermore, the electrocatalytic activity of Cu_3_N makes it a suitable electrode material for Li- and Na-ion batteries. Recent investigations have shown that Cu_3_N can also be integrated into microelectronic applications, i.e., for optical data storage when coupled with an Al_2_O_3_ protective layer and in spintronics when combined with FeN. In the thin film form, Cu_3_N presents promising attributes for the field of photovoltaics owing to its versatile band gap modified via the growth conditions in order to obtain higher optical absorption. Therefore, thin-film and nanoparticle growth of Cu_3_N offer promising routes for the next generation of applications. Additionally, given its versatility, combining Cu_3_N with other materials could augment its electronic, optical and photocatalytic properties. Extensively studied materials, such as carbon nanomaterials (graphene or CNTs), could enhance the adsorption of dyes and, in turn, photocatalytic properties of Cu_3_N. Carbon-based nanomaterials themselves serve as efficient dye adsorbents and, at the same time, enhance the charge conductivity of the inorganic material, thereby facilitating charge separation and redox reactions. Furthermore, they also tend to protect the inorganic material against oxidation, whereupon providing them structural and chemical stability. Therefore, by combining the above strategies, highly efficient, stable, low-cost and advanced semiconductors for large-scale photocatalysis could be fabricated. Developing a stable, high efficiency and cost-effective photocatalytic system will be the central theme of future research. Cu_3_N-based nanostructure research is clearly challenging and still young, but adding these nitrides to the portfolio of photocatalytic materials with excellent optical properties would open up many exciting possibilities for environmental remediation.

## Figures and Tables

**Table 1:** Summary of synthesis conditions and experimental band gaps for Cu_3_N nanostructures.

Cu_3_N Morphology	Experimental Details			Band Gap (eV)		Second Phases	Ref
	Synthesis Method	Precursors and Substrates	Conditions	Direct	Indirect		
Nanocubes	Single source precursor method	Cu(NO_3_)_2_ 3H_2_O, ODA	1. 110 °C, 1 h 2. 260 °C, 5 min	1.89	-	CuO	[24]
	Thermal decomposition	Cu(NO_3_)_2_⋅3H_2_O, OAm, ODE	1. 110 °C, 1 h 2. 210 °C, 15 min	-	1.6 eV	-	[38]
	Ammonolysis reaction	Cu(OAc)_2_, urea	1. 300 °C, 2 h	-	-	-	[46]
	One-phase process	Cu(NO_3_)_2_⋅3H_2_O, ODA (or HAD or OAm) + ODE	1. 150 °C, 3 h 2. 250 °C, 30 min	1.5	1.04	-	[47]
	One-step synthesis	Cu(NO_3_)_2_⋅3H_2_O, ODA	1. 115 °C, 1 h 2. 240 °C, 5 min	2.41	-	-	[48]
	Thermal decomposition	Cu(NO_3_)_2_⋅3H_2_O, ODA	1. 240 °C, 10 min	-	-	Cu/Cu_2_O	[49]
	Solvothermal synthesis method	Cu(NO_3_)_2_⋅3H_2_O, ODA, OAm	1. 110 °C, 1 h 2. 240 °C, 40 min	-	-	-	[50]
Spherical nanoparticles	Single source precursor method	PPC, ODA	1. 110 °C, 1 h 2. 260 °C, 5 min	2.21	-	Cu	[24]
	Pyridine-based synthesis	CuI, pyridine, NH_3_aq, KNH_2_	1. −35 °C 2. 130 °C, 30 min	2.0	-	-	[51]
	Thermal decomposition	Cu(NO_3_)_2_⋅3H_2_O, HAD	1. 110 °C, 1h 2. 230 °C, 5 min	2.92	-	Cu/CuO	[52]
	Ammonolysis reaction	Cu(CO_2_CH_3_)_2_ H_2_O, 1-nonanol, NH_3_ gas	1. 190 °C, 1h 2. 170 °C 3. 185 °C	-	-	CuO	[53]
	Surfactant-free Solution-phase approach	Cu(OMe)_2_, BZA	140 °C, 15 min	-	-	-	[54]
	Ammonolysis reaction	CuF_2_, NH_3_ gas	1. 140 °C, 6 h 2. 300 °C, 8 h	-	-	-	[55]
	Ammonolysis reaction	CuCO_3_, pivalic acid, NH_3_ gas	1. 70 °C, 30 min 2. 250 °C, 10 h	-	-	Cu	[55]
	Ammonolysis reaction	CuC_10_, 1-nonanol, NH3 gas	1. 190 °C, 40 min	-	-	-	[56]
Powders	Solid state reaction	CuO, NaNH_2_	1. 170 °C, 60 h	-	-	Cu/CuO	[57]
	Wet processing and ammonolysis	Cu(CF_3_COO)_2_, NH_3_ gas	250–350 °C, 45 min–5 h	1.48	-	Cu	[58]
	Solvothermal synthetic method	CuCl_2_, NaN_3_, Toluene/ solvent	1. ~50 °C, 4 h 2. ~100 °C, 10–12 h 3. T ↑ for several days: ~40 °C/day	-	-	CuO	[59]
	Ammonolysis reaction	CuF_2_, NH_3_ gas	250–350 °C, 6 h–18 h	-	-	-	[60]
Nanocrystals	Single-step solvothermal approach	Cu(NO_3_)_2_⋅5H_2_O, hexamethylenetetramine (HMT)	200 °C, 1 h	1.6	-	-	[26]
	PEALD	(Cu(hfac)_2_), NH_3_ plasma gas	ALD cycle: 2 s for Cu(hfac)_2_ (80 °C) in 0.5 Torr, 5 s for NH_3_ plasma, and 5 s of N_2_ purge at 1 Torr.	1.92	-	-	[34]
	Solution-phase synthesis	Cu(NO_3_)_2_⋅3H_2_O, ODE, OAm	1. 120 °C, 10 min 2. 240 °C, 15 min	-	-	-	[61]
	Ammonolysis reaction	Cu_2_O, NH_3_ gas	250 °C, 21 h	0.95	-	-	[62]
	Solvothermal process	Cu(NO_3_)_2_⋅3H_2_O, ODE, OAm or HDA	1. 150 °C, 3 h 2. 220 °C, 10 min 3. 250 °C, 10 min	-	-	Cu/Cu_2_O	[63]
Thin films	Thermal evaporation and ammonolysis reaction	Silicon substrate and ammonia solution	1. 120 °C, 1 h 2. 180 °C, 2 h 3. 310 °C, 4 h	2.0	-	Cu/Cu_2_O	[28]
	RF and magnetron sputtering	On silicon slice and quartz plate substrate	P = 1.0 Pa, N_2_ gas flow 40 sccm, RF power 300 W	1.23 −1.91		-	[25]
	DC magnetron sputtering	Quartz glass substrates	P = 1.0 Pa, N_2_ gas flow 3.5–4.0 sccm, RF power 100–130 W.	-	1.44	-	[64]
	Magnetron sputtering	Single-crystal silicon and quartz substrates	P = 1.0 Pa, N_2_ gas flow 40 sccm, RF power 250 W.	2.0	-	Cu/Cu_2_O	[65]
	Modified activated reactive evaporation	Borosilicate glass substrate	P = 10 mTorr, N_2_ gas flow 40 sccm, RF power 50 W	2.15	1.60	-	[66]
	Thermal evaporation method	Glass substrate	1. P = 10^−2^ Torr, 1000 °C 2. N_2_ gas flow 100 sccm, 300 °C	-	-	Cu	[67]
	RF reactive sputtering	Glass substrate	P = 10 mTorr of nitrogen balanced by 10 mTorr of argon, 140–280 °C	-	-	-	[68]
Doped Cu_3_N and their nanocomposites							
Cu_3_N:Pd films	RF and DC magnetron sputtering	Single-crystal silicon substrate	P = 2 × 10^−3^ Pa, Ar gas flow 10 sccm, N_2_ gas flow 30 sccm, RF power 200 W, DC power 0–7 W for Pb	-	-	-	[30]
Cu_3_N:Ag Thin film	RF and DC magnetron sputtering	Monocrystalline silicon and glass substrate	P = 10 × 10^−3^ Pa, N_2_ gas flow 40 sccm, RF power 200 W	-	1.59	-	[69]
Cu_3_N nanocrystals on CNTs	PEALD	(Cu(hfac)_2_), NH_3_ gas	P = 1 torr, plasma power 100–400 W, NH_3_ gas 250 °C	1.9	-	-	[70]
Cu_3_N@SiO_2_ spheres	Ammonolysis	CuSiO_3_, NH_3_ gas	350 °C, 1 h	-	-	-	[71]

Acronyms: Octadecene (ODE), 1-octadecylamine (ODA), hexadecylamine (HAD), oleylamine (OAm) and benzylamine (BZA), PPC = pyrrole-2-carbaldpropyliminato Cu(II).

**Table 2:** Summary of photocatalytic behavior of Cu_3_N-based nanomaterials.

Semiconductor Composite	Band Gap (eV)	Dye Concentration	Condition	Degradation Time	Efficiency	Particle Size	Ref
Cu_3_N@MoS_2_ thin films	2.05	MO–10 mg/mL	500 W Hg–lamp	30 min	98.3%	-	[35]
Au-decorated Cu_3_N nanocubes	1.67	MO/MB 0.0008 mM	250 W Universal arc lamp	25 min for MB and MO	84% for MO 93% for MB	NC: 10 ± 5 nm Au: 10 and 5 nm	[38]
Cu_3_N nanocubes	2.41	MO/MB 20 ppm	Solar simulator source 100 mW/cm^2^	180 min for MO 240 min for MB	89% for MO 61% for MB	NC: 41 ± 7 nm	[48]
Cu_3_N thin films	2.0	MO–20 mg/L	500 W Hg–lamp	30 min	95.5%	-	[65]
TiO_2_@Cu_3_N thin films	3.0	MO–20 mg/L	500 W Hg–lamp	30 min	99.2%	-	[84]
